# An Analysis of Multilevel Barriers to Human Papillomavirus Vaccination Uptake Among Rural U.S. Adolescents

**DOI:** 10.3390/vaccines14090772

**Published:** 2026-09-02

**Authors:** Tajauna Batchelor, Madison Brown, Kimbrionna Hunter, Asma Hanif, Shumaila Nida Javed Tunio

**Affiliations:** 1 Department of Biomedical Sciences, University of Pikeville, Pikeville, KY 41501, USA; 2 Department of Restorative Sciences, College of Dentistry, Kuwait University, Kuwait City 12037, Kuwait; 3 Lake Eire College of Osteopathic Medical School, 5415 Dolphin Point Blvd, Jacksonville, FL 32211, USA

**Keywords:** HPV vaccination, barriers, differences, adolescents, provider-recommendation, rural communities

## Abstract

Despite longstanding vaccine availability, human papillomavirus (HPV) remains the most common sexually transmitted infection in the United States and a leading cause of preventable cancers. Additionally, HPV vaccination rates remain below other routinely recommended adolescent immunizations, particularly in rural populations. This study aimed to identify barriers related to healthcare access, socioeconomic conditions, cultural beliefs, and provider–patient communication in rural communities. A bibliographical review of articles published in English from 2020 to 2025 and an analysis of national datasets were conducted to establish trends in HPV vaccination rates. State-level HPV vaccination data for adolescents aged 13–17 years were obtained from America’s Health Rankings and the Centers for Disease Control and Prevention National Immunization Survey-Teen. States were classified as predominantly rural or urban using Rural–Urban Continuum Codes, and mean vaccination completion rates were compared. The mean HPV vaccination completion rate was lower in rural states (60.55%) compared to urban states (67.31%); however, this difference did not meet the selected threshold for statistical significance (*p* = 0.025). Barriers identified in the literature included reduced access to healthcare, differences in provider communication, socioeconomic constraints, and limited health literacy in rural communities. Of the identified barriers, healthcare provider recommendations emerged as one of the strongest predictors of vaccine acceptance. These findings highlight multilevel determinants contributing to differences in HPV vaccine uptake and underscore the need for targeted, evidence-based strategies to improve vaccine access and coverage in underserved adolescent populations.

## 1. Introduction

Human papillomavirus (HPV) is the most common sexually transmitted infection (STI) worldwide. It is responsible for a substantial proportion of cancers, including cervical cancer, which is the second leading cause of cancer mortality among women [1]. This global burden is also reflected in the United States, where HPV causes 91% of cervical and anal cancers, 71% of oropharyngeal cancers, and 69% of genital cancers [1,2,3]. Currently, there are over 200 HPV strains, with strains 16 and 18 accounting for the majority of HPV-related malignancies [4]. In contrast, low-risk HPV strains, most often 6 and 11, can cause visible growths on the skin and mucous membranes, including common and genital warts, which are typically benign [3,5]. Given the significant health risks associated with HPV, vaccination provides safe, effective, and durable protection against high-risk viral strains. The Centers for Disease Control and Prevention (CDC) recommends routine vaccination at ages 11 to 12 and permits initiation as early as 9 years [6,7,8,9]. Consistent with this administration schedule, research demonstrates a stronger immune response when vaccination is initiated in childhood [8].

The HPV vaccine developed from parallel breakthroughs by scientists across the globe. The starting factor in this research was by Dr. Harald zur Hausenwas, who hypothesized that there was a link between human papillomaviruses and cervical cancer after observing papillomaviruses cause cancer in animals [10]. Years later, in 1991, Drs. Ian Frazer and Jian Zhou developed virus-like particle (VLP) technology, which mimics the outer shell of HPV and triggers an immune response without the need for viral DNA [10]. This discovery allowed researchers, including Drs Dough Lowy and John Schiller, to develop the HPV vaccine using the VLPs. The first HPV vaccine, Gardasil 4, was approved in 2006 for females only, and specifically targeted HPV types 6, 11, 16, and 18 [11]. Three years later, the vaccine was approved for use in males, and alternative vaccines were created. One of these was Cervarix, which only targeted strains 16 and 18; however, it was pulled from the market in 2016 following the success of Gardasil 9. Gardasil 9, released in 2014 and the main vaccine used currently, offers protection from low-risk wart-causing HPV strains, like strains 6 and 11, as well as high-risk cancer-causing strains, specifically strains 16, 18, 31, 33, 45, 52, and 58 [11].

Despite the proven benefits of HPV vaccination, full coverage among U.S. adolescents remains below national standards. In 2022, approximately 76% of adolescents aged 13 to 17 received at least one dose of the HPV vaccine, and 62.6% completed the full two-dose series, falling short of the Healthy People 2030 goal of 80% series completion [12,13]. Although national coverage appears sufficient, geographic differences persist. Specifically, adolescents in rural areas consistently have lower vaccination rates than their urban counterparts, reflecting multilevel barriers in rural communities. [12,14].

Reducing differences in HPV vaccination is critical for promoting public health equity and preventing avoidable HPV-related cancers in underserved populations. Most studies focus on HPV vaccine uptake through a broad geographical lens with little attention to the unique barriers faced by adolescents in rural areas. Although general strategies to increase vaccination uptake have been identified, there is limited evidence regarding how well these approaches translate to improving HPV vaccination rates in rural communities [15,16]. This gap emphasizes the need for research examining how contextual factors influence HPV vaccine uptake in marginalized populations. Therefore, this study examines multilevel factors that impact HPV vaccine trends in rural adolescents and aims to provide insight into areas where public health efforts may be most impactful.

## 2. Materials and Methods

### 2.1. Design

A narrative review examining perceived barriers and facilitators contributing to differences in HPV vaccination uptake was conducted. Articles evaluating barriers to access and preventive strategies were collected to establish a comprehensive knowledge base for this review. Zotero was used to manage and organize all articles, facilitating shared access among co-authors and ensuring systematic screening and consistency throughout the review process. In addition to the literature review, statistical and descriptive analyses of HPV vaccination trends in rural and urban settings were performed.

### 2.2. Eligibility Criteria

The inclusion criteria consisted of primary studies published in English from 2020 to 2025 that analyzed data from the United States and focused on adolescents aged 9 to 17 years and examined HPV vaccination uptake, with particular attention to rural populations or geographic differences.

### 2.3. Information Sources

Articles were identified through searches of PubMed and the National Library of Medicine databases. Combined data from the Centers for Disease Control and Prevention National Immunization Survey-Teen (CDC NIS-T), America’s Health Rankings, and Rural–Urban Continuum Codes (RUCC) were utilized for descriptive analysis [13,17,18]. The CDC NIS-T provided vaccination status data for adolescents ages 13 to 17 across the U.S., as well as a standardized definition of ‘full vaccination status’ for this review [13]. America’s Health Rankings provided state-level vaccination completion rates for adolescents aged 13 through 17 in 2023 [17]. Finally, RUCC classified U.S. counties as rural or urban based on population characteristics [18].

#### 2.3.1. Background on Datasets

The NIS-Teen survey provides data to guide public health efforts aimed at improving HPV vaccination completion rates in adolescents. This survey uses random-digit dialing to contact households with teenagers aged 13 to 17 years about vaccination histories, followed by verification with the adolescents’ primary care providers. Full vaccination status was defined as completion of the two-dose HPV vaccine series recommended by the CDC when the first dose is initiated prior to a teenager’s 15th birthday [19].

State-level HPV vaccination completion rates were obtained from America’s Health Rankings, which analyzed NIS-Teen data collected in 2023 and compiled the findings into state-level vaccination completion rates for adolescents aged 13–17 years [13,17].

For the descriptive analysis portion of this project, a standardized definition of ‘rural’ and ‘urban’ was used to accurately characterize the distribution of rural and urban communities within each state. The RUCC classification system, developed and maintained by the USDA Economic Research Service, was used for this project. The RUCC are updated every decade, with the most recent revision in 2023. These codes classify counties into two categories: metropolitan (metro) and nonmetropolitan (nonmetro). Metro counties are defined as those located within labor-market areas with populations of 50,000 or more, including surrounding counties that have economic ties to the urban areas, measured through commuting patterns. Nonmetro counties are classified according to population size (20,000 or more, 5000 to 20,000, and fewer than 5000) and proximity to a metro area, defined as physically adjacent to a metro county and at least 2% of the county’s workforce commuting to metro counties for work. According to the 2023 RUCC, there were 1186 metro counties and 1958 nonmetro counties across all fifty states [18].

Important note: data from 2023 was utilized for the analysis due to that being the most up-to-date data at the time of analysis.

#### 2.3.2. Statistical Analysis

An independent *t*-test was performed to compare the dependent variable of HPV completion rates between the independent variable of state classification using Statistical Package for the Social Sciences (IBM), version 31. Given the potential for Type 1 error, a conservative significance threshold was established, with *p* < 0.01 was considered statistically significant.

### 2.4. Search Strategy and Research Question

Keywords such as “HPV vaccination,” “barriers,” “differences,” “adolescents,” “provider-recommendation,” “rural communities,” and a combination of these keywords were used to collect articles for review. The review had the following research question and objectives:

Primary research question:

What barriers contribute to lower HPV vaccination rates in rural adolescents, and what strategies can improve uptake?

Objectives:

1. Identify and evaluate barriers that affect HPV vaccination status in rural communities.
2. Identify interventions aimed at improving HPV vaccination uptake and summarize reported outcomes.

### 2.5. Selection Process

The selection process for this review followed a two-phase approach: an initial collection and screening of abstracts, followed by an in-depth review of eligible full-text articles. All potentially relevant articles were uploaded to Zotero. Each author independently reviewed the selected articles to ensure a comprehensive analysis and to identify key study characteristics. Any discrepancies between reviewers were resolved through discussion and consensus.

## 3. Results

A total of 31 articles met the inclusion criteria for this review. Results are represented as descriptive and statistical findings from national datasets and thematic findings from the literature review.

### 3.1. Statistical and Descriptive Analysis of HPV Vaccination Coverage

#### 3.1.1. HPV Vaccination Uptake Compared with Other Adolescent Immunizations

**Figure 1:**
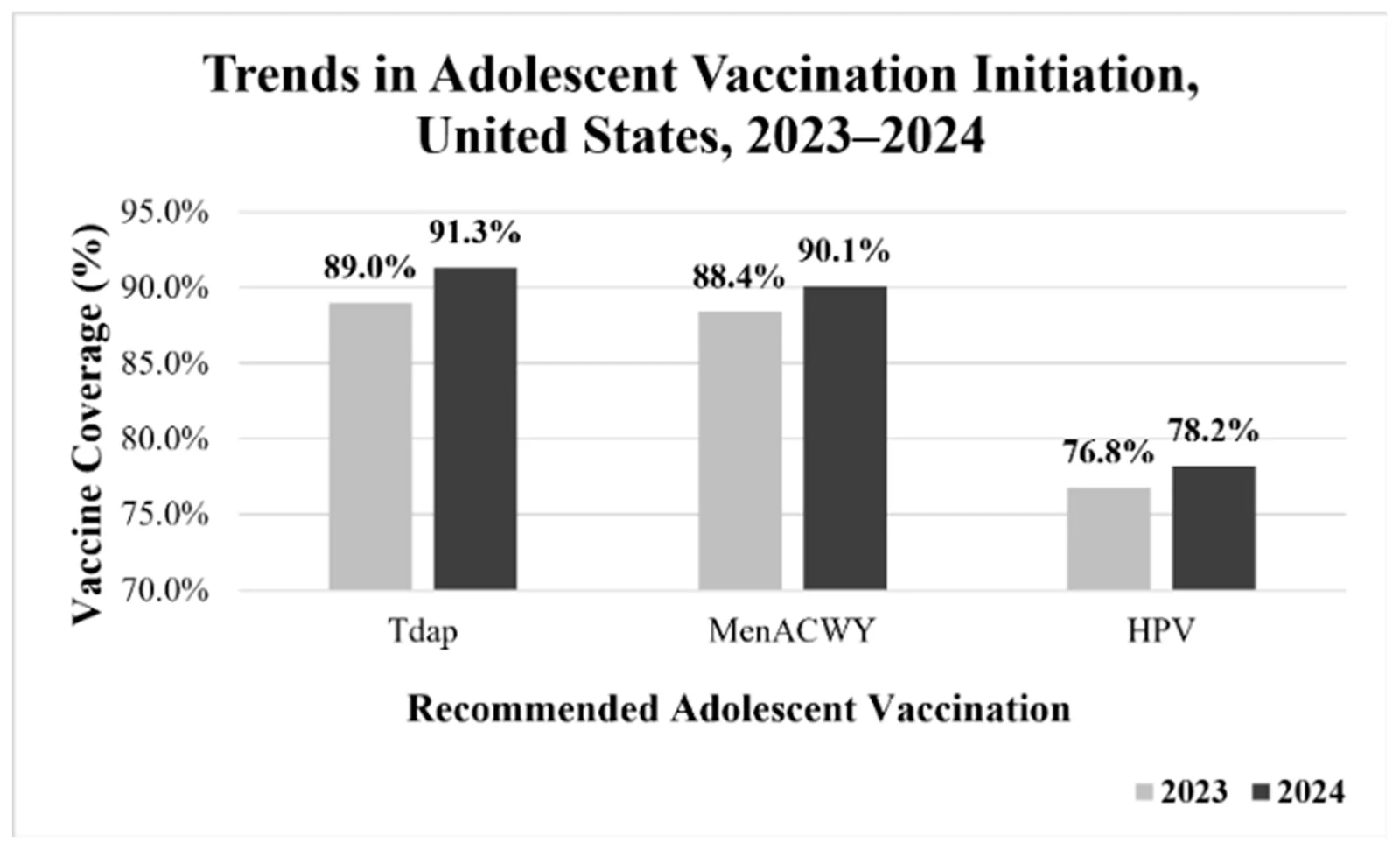
National vaccination initiation rates (≥1 dose) for routinely recommended adolescent vaccines (ages 13–17 years) in the United States for 2023 and 2024. Data were obtained from the CDC NIS–Teen. Vaccines included Tdap, MenACWY, and HPV. Percentages represent national vaccination initiation rates [13].

Vaccination initiation status (≥1 dose) for routinely recommended adolescent vaccines, including tetanus–diphtheria–acellular pertussis (Tdap), meningococcal conjugate (MenACWY), and HPV, was obtained from the NIS-Teen survey to evaluate HPV vaccine uptake relative to other adolescent immunizations. National initiation percentages from 2023 and 2024 were compiled into Figure 1. Tdap and MenACWY consistently demonstrated the highest overall initiation rates, exceeding 88% each year, while HPV vaccination remained below 80% in both years [13].

#### 3.1.2. State-Level HPV Vaccination Completion in Rural and Urban States

**Figure 2:**
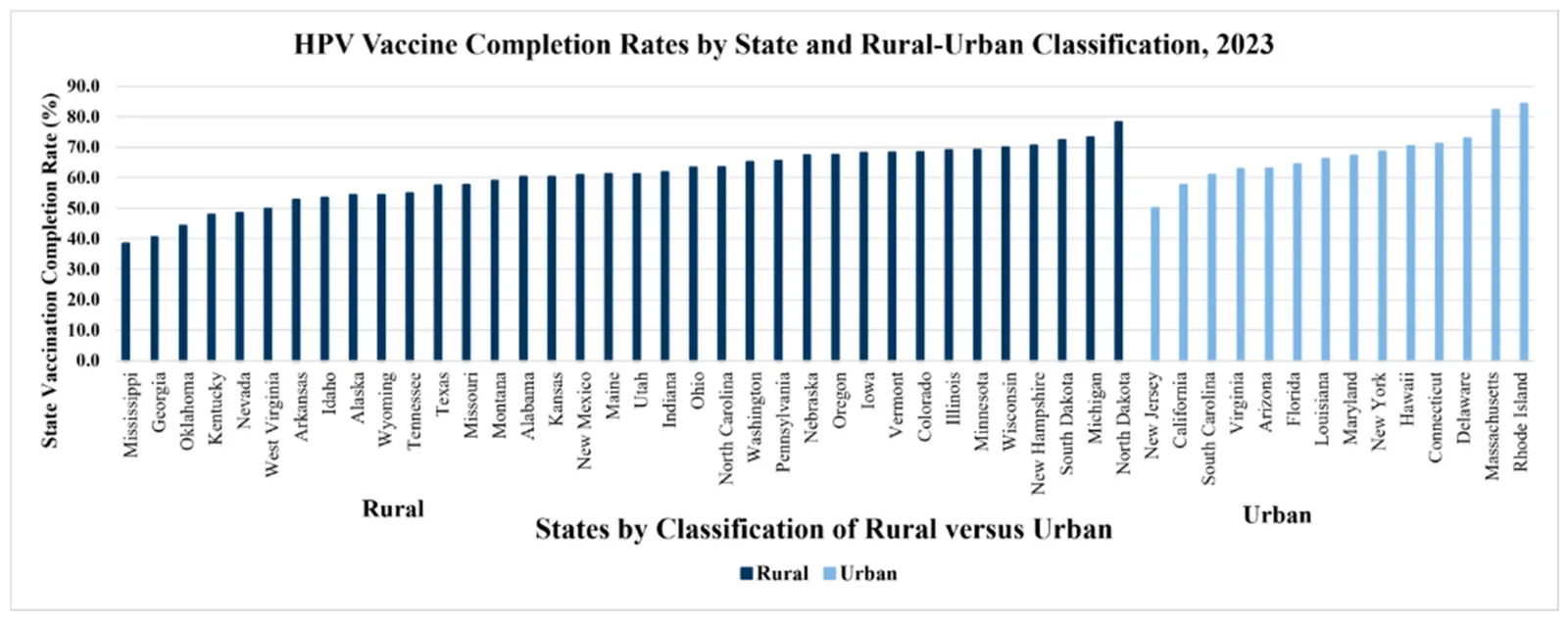
State-level HPV vaccination completion (≥2 doses) for adolescents aged 13–17 years in the United States from 2023. Data were obtained from NIS-Teen and compiled by America’s Health Rankings. States were classified as predominantly rural or urban using 2023 RUCC criteria; states with more than 50% of counties designated as nonmetropolitan were deemed rural. Bars represent state-level vaccination rate percentages [13,17,18].

HPV vaccination completion (≥2 doses) data from NIS-Teen and America’s Health Rankings were compiled into Figure 2 to compare rural and urban vaccination rates. Rural states demonstrated greater variability and generally lower completion rates, with several states being below 60%. In contrast, urban states exhibited higher completion rates, with several states exceeding 70%, suggesting a pattern of rural–urban differences in HPV vaccine coverage [13,17,18].

#### 3.1.3. Mean HPV Vaccination Completion Rates by Rural–Urban Classification

**Figure 3:**
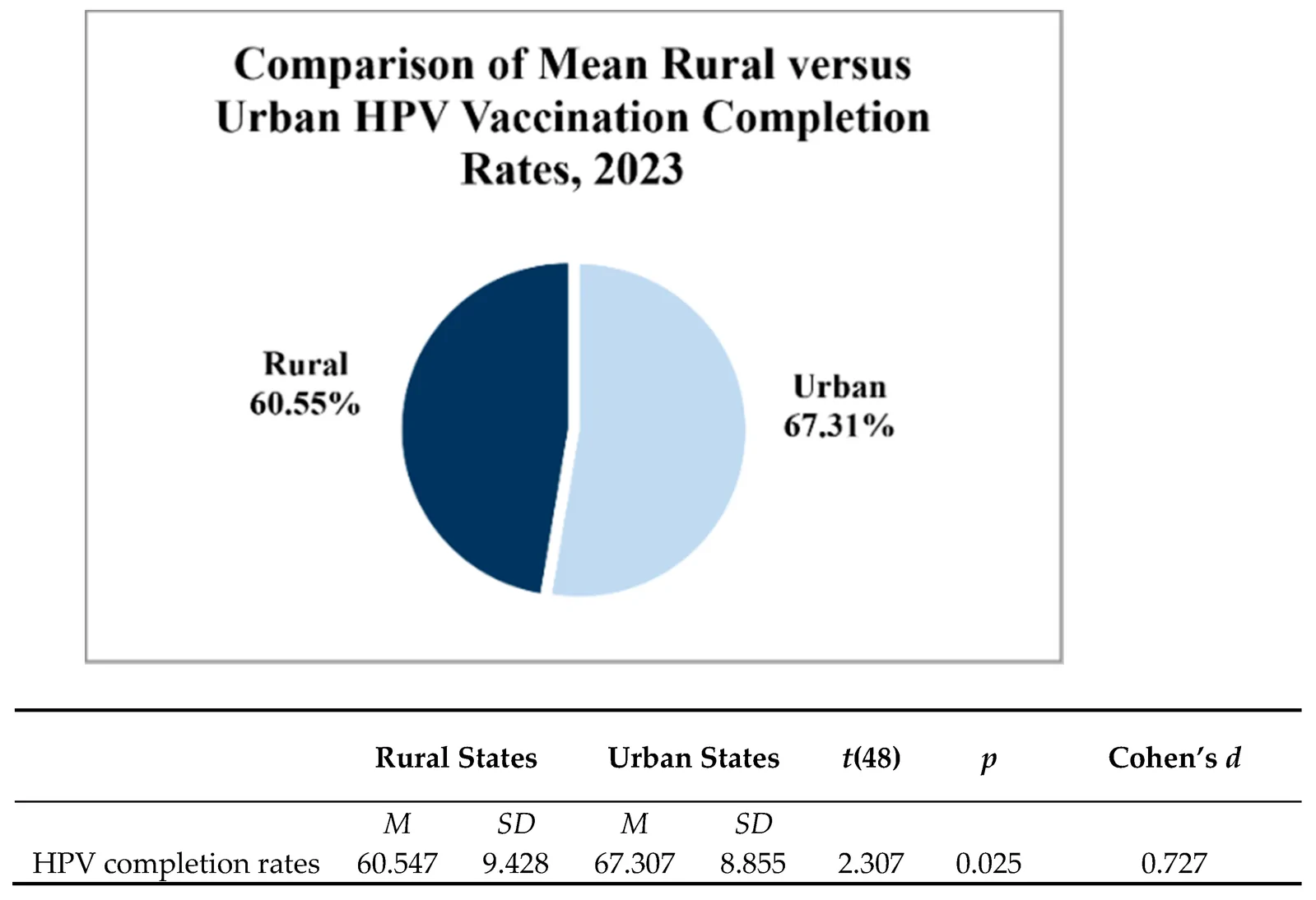
Mean state-level HPV vaccination completion (≥2 doses) for adolescents ages 13–17 in the United States from 2023. Values represent mean completion rate across states within each classification [13,17,18]. An independent *t*-test was performed to compare the dependent variable of HPV completion rates between the independent variable of State classification using Statistical Package for the Social Sciences (IBM), version 31. To reduce the risk of reporting that the findings were significant when in fact they occurred by chance (Type I error), *p* < 0.01 was considered statistically significant.

Figure 3 presents mean HPV vaccination completion rates across rural versus urban states. Rural states had a lower mean average completion rate (60.55%, SD = 9.43) compared with urban states (67.31%, SD = 8.86), representing a 6.76 percentage point difference in mean completion rates [11,15,16]. An independent *t*-test was conducted to assess whether HPV vaccination completion rates differed between rural and urban states. The difference did not meet the preselected threshold for statistical significance (t = 2.307, *p* = 0.025, *p* < 0.01). However, the observed effect size was moderate to large (Cohen’s d = 0.727), indicating there is a meaningful magnitude of difference between rural and urban mean completion rates.

### 3.2. Identified Barriers to HPV Vaccination in Rural Adolescents

#### 3.2.1. Sociocultural Barriers

Several studies recognized sociocultural influences like parental education level, religious beliefs, and sexual health stigmas as key contributors to HPV vaccine hesitancy in rural populations [20,21,22,23]. Many of these studies reported that caregivers in rural communities, including the rural U.S. South, expressed concerns that HPV vaccination may be perceived as condoning sexual activity or require conversations about sex that conflict with prevailing social or religious norms [21,24]. For example, in a qualitative study of 40 participants in rural southwest Georgia, including parents of vaccinated and unvaccinated adolescents, young adults, and healthcare and public health professionals, described the association between HPV and sexual activity as a source of stigma and discomfort surrounding vaccination [22,25]. Religious and abstinence-oriented beliefs also influence perceptions of vaccine necessity, with concerns that vaccination may conflict with prevailing social norms [22]. Similarly, interviews with 14 public health, education, and community stakeholders across rural North and South Carolina identified negative community attitudes and concerns surrounding sexual activity as barriers to HPV vaccination, although some stakeholders perceived these concerns as becoming less prominent over time [20]. 

Survey findings across 13 Southern states further support the role of caregiver attitudes in rural HPV vaccination differences despite similar overall knowledge [21]. The results revealed rural caregivers reported less favorable perceptions of vaccine benefits and social support for vaccination [21]. National data also demonstrated sex-specific differences in parental reasons for lacking intent to initiate HPV vaccination, suggesting that perceptions of vaccine necessity and acceptability may vary according to adolescent sex [23]. Together, these findings suggest that sociocultural barriers extend beyond insufficient knowledge and may reflect broader community values and perceptions that shape caregiver acceptance of HPV vaccination in rural settings.

#### 3.2.2. Knowledge and Awareness Barriers

Limited awareness of HPV-related disease and vaccination emerged as another barrier to HPV vaccine uptake in rural populations [20,21,22,26,27,28]. Studies identified limited knowledge of HPV and HPV vaccination among parents and adolescents, alongside gaps in community awareness and access to vaccine information as key contributing factors to differences in rural–urban vaccination rates [20,22]. Interviews with community stakeholders specifically described parental concerns about vaccine safety and adverse effects, including misinformation encountered through social media, and emphasized limited access to comprehensive sexual health education in rural communities [20]. Additionally, limited broadband connectivity was also described as a constraint on disseminating reliable vaccine information to rural families, further restricting opportunities for education [20]. These regional observations align with broader evidence identifying inadequate HPV and vaccine knowledge as recurring obstacles to vaccination program implementation [26].

However, knowledge deficits alone may not fully explain rural–urban differences in HPV vaccination. In a survey of 987 caregivers across 13 Southern states, including 193 rural caregivers, overall HPV knowledge scores were similar between rural and urban responders [21]. Nonetheless, specific gaps remained as rural caregivers were less likely to recognize HPV as a cause of head and neck cancers or understand the high lifetime prevalence of HPV infection [21]. Broader vaccine hesitancy may further compound these concerns. Among 27 East Texas clinics, 33.3% identified HPV vaccine-specific hesitancy as a barrier, while 44.4% reported vaccine hesitancy associated with the COVID-19 pandemic [28]. Collectively, these findings suggest that improving HPV knowledge remains important, but information-based interventions alone may be insufficient when misinformation, safety concerns, and limited access to trusted health information influence vaccine decision-making [20,21,22,28].

#### 3.2.3. Structural Barriers

Geographic and socioeconomic constraints can reduce opportunities for rural adolescents to receive preventative services and complete HPV vaccination [20,22,29,30]. Across the literature, transportation difficulties, greater travel distances, healthcare workforce shortages, insurance status, and financial burdens emerged as interconnected obstacles to vaccine delivery. These challenges may be particularly consequential for HPV vaccination because inadequate access to routine and follow-up care can result in missed opportunities for both series initiation and completion.

National data further illustrate the intersection of these burdens and how they are experienced within rural communities. In the 2020 NIS-Teen, HPV vaccination initiation among adolescents living in rural areas was 68.0%, compared to 77.8% among those living in metropolitan principal cities; corresponding up-to-date coverage was 49.2% and 60.4%, respectively [29]. Among adolescents at or above the federal poverty level, up-to-date coverage was 13.8 percentage points lower in nonmetropolitan areas than metropolitan principal cities, whereas comparable geographic differences were not statistically significant among adolescents living below the federal poverty level [29,31]. More recent multilevel analyses similarly found that greater Medicaid eligibility, public insurance coverage, and access to pediatricians and Vaccines for Children (VFC) providers were positively associated with both HPV vaccine initiation and completion. Specifically, each additional pediatrician or VFC provider per 100,000 children was associated with a 0.01 percentage-point increase in both initiation and completion [31]. Notably, rurality was not independently associated with initiation after accounting for these demographic and policy factors.

County-level data further reinforce the importance of considering structural factors in combination rather than treating rural residence as an isolated determinant. In Michigan, gains in HPV vaccination completion among male adolescents from 2017 to 2023 were smaller in counties with lower household income and higher uninsured rates, whereas differences according to rurality were inconsistent [30]. Taken together, these findings suggest that rural HPV vaccination differences arise from overlapping geographic, financial, and healthcare-system constraints that can reduce opportunities for preventative care rather than rural residence alone [20,22,29,30].

#### 3.2.4. Healthcare Provider and Delivery Level Barriers

Healthcare providers (HCP) play a central role in vaccine uptake, with the strength and consistency of provider recommendation repeatedly associated with vaccination [22,24,32,33,34]. However, opportunities for recommendation are not equally distributed across populations. National analyses demonstrate geographical and regional variation in parental report of provider recommendation, with adolescents in the South having lower odds of receiving a recommendation than those in the Northeast [34]. Recommendation patterns also varied by patient characteristics, including age and sex, suggesting that inconsistent communication may contribute to missed opportunities among populations already experiencing lower vaccination coverage [24,34].

Studies further illustrate how HCP communication can impact vaccine uptake, highlighting the importance of communication and trust. In rural southwest Georgia, parents and adolescents described HCP recommendation and trusted relationships with their provider as influential in vaccination decision-making [22]. Yet clinicians may face difficulty navigating vaccine hesitancy while communicating HPV vaccination as routine preventative care. Provider knowledge may also affect communication, with surveys of HCPs identifying variability in HPV vaccine knowledge and recommendation practice across professional groups [33]. Consequently, the effectiveness of the clinical encounter depends not only on whether vaccination is discussed, but also on the consistency, confidence, and framing of the recommendation. Interviews with rural-and non-rural serving HCPs found that presumptive recommendations could normalize HPV vaccination and facilitate uptake, but parental resistance, limited visit time, and concerns about maintaining trust could complicate consistent implementation [35].

Additionally, delivery challenges extend beyond HCP communication to the infrastructure required to reliably administer and complete vaccination [20,25,27,28,31,35]. Rural practices have reported staffing constraints, competing clinical priorities, inconsistent reminder and recall systems, and variable implementation of evidence-based vaccination strategies as provider-level barriers that impact clinic efficacy, particularly surrounding vaccine uptake [20,28]. Rural South Carolina pharmacists similarly identified reimbursement, vaccine storage and supply, workflow demands, staff training, and limited patient demand as obstacles to expanding vaccine delivery [25]. Although pharmacies and community-based partnerships may broaden vaccination opportunities, their implementation remains dependent on adequate staffing, financing, coordination, and integration with existing healthcare systems [25,27,35]. Therefore, HCP and delivery-level factors may represent a critical bottleneck across the vaccination pathway: even when knowledge, acceptance, and vaccine availability are present, inconsistent recommendations or insufficient delivery capacity can prevent these conditions from translating into vaccine initiation and completion [24,28,31,32] (Table 1).

## 4. Discussion

Despite the availability of an effective vaccine and rising completion rates, HPV vaccination remains below national targets, with a persistent gap between rural and urban populations. Across the literature, HPV vaccination rates appear to be shaped by interacting structural, sociocultural, and provider-level factors. One of the strongest predictors of HPV vaccination uptake noted was HCP recommendation, suggesting provider engagement may serve as a key modifiable mediator to improving vaccine access and acceptance [31]. This review synthesized existing evidence to identify barriers to HPV vaccination among rural adolescents and highlights strategies to improve uptake, with a particular focus on the role of healthcare providers in addressing multilevel determinants.

Our state-level analysis demonstrated a lower mean HPV vaccination completion rate among predominantly rural states when compared to predominantly urban states. Although this difference did not reach statistical significance, the observed effect size was moderate-to-large (Cohen’s d = 0.727), suggesting a potentially meaningful difference in vaccination completion between the two groups, despite the lack of statistical significance.

In this multilevel context, structural barriers constrain vaccination opportunities in rural communities. Compared with urban adolescents, rural youth often face reduced healthcare access and fewer provider interactions, which limit exposure to vaccine recommendations. Additionally, reduced HCP recommendation rates in rural and more socially conservative areas likely reflect overlapping provider and system-level challenges, including workforce shortages, competing clinic priorities, and limited training in vaccine communication. Thus, provider recommendation functions as a critical intermediary point between structural access constraints and individual vaccine uptake, allowing for tangible interventions within deeply integrated system-level barriers.

In rural communities, limited access to reliable healthcare resources may increase exposure to misinformation regarding vaccine safety and efficacy. However, informational gaps do not operate in isolation; they interact with prevailing sociocultural norms to shape perceptions of HPV vaccination. Because the vaccine is frequently framed through a reproductive health lens, caregivers in socially conservative settings may interpret vaccination as unnecessary or morally misaligned, particularly in the context of abstinence-based beliefs [14,25,26]. As a result, vaccine decision-making becomes embedded within broader cultural narratives, shaping how provider recommendations are interpreted and acted upon.

Strong and strategically framed provider recommendations may counter both misinformation and vaccine-related stigma. By presenting the HPV vaccine as a cancer prevention strategy rather than a sexual health intervention, providers can align vaccination with broader health priorities and facilitate informed caregiver decision-making. Narrative-based approaches and partnerships with community-based organizations have shown promise in reshaping views toward the vaccine, while school-based programs can improve uptake by reaching adolescents during periods of declining primary care visits [22,30,36,37]. Additionally, studies further support enhanced provider education and communication training, including the use of presumptive language, motivational interviewing, and multi-language hand-outs as intervention strategies [30,32,38,39]. Moreover, early and frequent discussions before adolescent visits may provide anticipatory guidance, allowing caregivers more time to engage with information [40]. These approaches may be particularly important in communities where abstinence-based beliefs influence perceptions of vaccine necessity; however, these efforts may be less effective in settings where healthcare infrastructure constraints and misconceptions remain unaddressed.

Collectively, these findings highlight the interdependence of structural access, sociocultural framing, and provider communication in HPV vaccination differences. Accordingly, consistent, clearly framed provider recommendations that position the vaccine as routine preventive care, coupled with targeted provider education and communication training, represent high-yield opportunities to increase coverage. Also, complementary public health messaging that reinforces this framing may further reduce stigma and support equitable vaccine uptake across diverse communities [41]. Ultimately, multilevel, context-specific interventions are essential to bridging rural–urban gaps and improving equitable HPV vaccine coverage.

## 5. Limitations

This review has several limitations. As a structured narrative review, findings are dependent on the quality and heterogeneity of included studies, which may vary in methodology and population characteristics. Additionally, the use of secondary data limits the ability to establish causality or account for all potential confounding factors. Because analyses were conducted using compiled state-level data, findings may not fully reflect individual state-level vaccination behaviors and may obscure substantial intra-state variability in rural and urban populations. Despite these limitations, this review provides a comprehensive synthesis of multilevel factors contributing to rural–urban differences in HPV vaccination.

## 6. Conclusions

This review underscores the need for multilevel, culturally responsive strategies to increase HPV vaccination uptake in rural adolescent populations. Addressing misinformation, improving HCP training, and reframing HPV vaccination as a universally relevant cancer prevention tool may help reduce persistent rural–urban coverage gaps and advance equitable vaccination uptake. Additionally, empowering healthcare professionals to consistently recommend HPV vaccination may represent an important practical opportunity to support vaccination uptake. Furthermore, the recently adopted single-dose schedule may further facilitate vaccination by reducing the number of healthcare visits required to complete the series, a consideration that may be particularly relevant in rural settings [42]. These efforts may ultimately enhance vaccine schedule adherence, thereby enhancing protection against HPV-associated malignancies nationwide.

## Figures and Tables

**Table 1:** Summary of multilevel barriers influencing HPV vaccination uptake among rural U.S. adolescents identified through the literature review. Barriers were categorized according to the primary level at which they influence vaccine decision-making and access. Healthcare provider communication was the most frequently identified barrier, followed by structural and socioeconomic barriers, knowledge and awareness gaps, and sociocultural influences.

Barrier 4.	Barrier Category	Studies Identifying Barrier (n)	Representative Examples	Proposed Impact on Vaccination Uptake
Interpersonal	Sociocultural Influences	4	Parental hesitancy, religious beliefs, stigma surrounding discussions of sexual health, community norms regarding vaccination	Reduced vaccine acceptance and delayed vaccination decisions
Individual	Knowledge and Awareness Gaps	7	Limited HPV knowledge, misconceptions regarding vaccine safety and effectiveness, low health literacy, lack of awareness of vaccination recommendations	Increased vaccine hesitancy and reduced perceived benefit of vaccination
Healthcare System	Structural and Socioeconomic Barriers	8	Transportation challenges, geographic isolation, provider shortages, limited insurance coverage, lower household income	Reduced access to preventive healthcare services and vaccination opportunities
Provider	Healthcare Provider Communication	12	Weak or absent recommendations, inconsistent messaging, limited counseling time, missed opportunities during clinical encounters	Lower vaccine initiation and completion rates despite healthcare access

## Data Availability

No new data were created or analyzed in this study. Data sharing is not applicable to this article.
